# Evaluation of eazyplex^®^ SuperBug CRE Test for Beta-Lactamase Genes Detection in *Klebsiella* spp. and *P. aeruginosa* Strains

**DOI:** 10.1007/s00284-019-01806-5

**Published:** 2019-11-14

**Authors:** Alicja Sękowska, Tomasz Bogiel, Eugenia Gospodarek-Komkowska

**Affiliations:** Department of Microbiology, Ludwik Rydygier Collegium Medicum, Nicolaus Copernicus University, 9 M. Sklodowska-Curie Street, 85-094 Bydgoszcz, Poland

## Abstract

The multi-drug resistance of Gram-negative rods is one of the most important issues of present medicine. In recent years, more and more strains resistant to the majority or to all possible therapeutic options have been isolated—especially *Klebsiella* spp. and *Pseudomonas* spp. representatives. It is very important to detect strains with these phenotypes as quickly and reliably as possible. The aim of the study was to evaluate the usefulness of eazyplex^®^ SuperBug CRE test (Amplex Diagnostics) for the detection of the most important beta-lactam resistance genes. eazyplex^®^ SuperBug CRE test is based on the loop-mediated isothermal amplification (LAMP) method, and detects genes for the following beta-lactamases: KPC, NDM-1, VIM, OXA-48, CTX-M1, CTX-M9 and OXA-181. The study involved 87 strains. For all of the positive strains in the LAMP method, additional PCR were performed to increase the spectrum of ESBLs detected by the genes encoding for enzymes belonging to TEM and SHV families. The results obtained by the tested method and standard PCR were consistent for all *Klebsiella* spp. strains. The discrepancy between the evaluated test and PCR results was observed for one *P. aeruginosa* strain. The eazyplex^®^ SuperBug CRE test can be used for quick detection of the most important beta-lactam resistance mechanisms amongst Gram-negative rods.

## Introduction

Multi-drug resistance of Gram-negative rods, including resistance to carbapenems, is one of the most important problems associated with the infections treatment. Carbapenems are often one of a very few or the only therapeutic option in case of the diseases caused by the strains producing beta-lactamases, especially Extended-Spectrum Beta-Lactamases (ESBLs). An unreasonable usage of carbapenems has led to increased isolation frequency of the strains with reduced susceptibility or completely resistant to this group of antibiotics. Time to detect the mechanism of carbapenem resistance is very important due to the rapid spread of the strains with such phenotype and their high virulence potential. In cases of colonization or infection of the patient with carbapenemase-producing strain, it is often necessary to isolate the patient or implement additional medical procedures that influence the total cost of hospitalization. Up till now, despite the high specificity and sensitivity of the test, and very short time to get the result of beta-lactamase resistance genes, there is only a few publications describing the use of eazyplex^®^ SuperBug CRE test in the available literature.

The aim of the study was to assess the usefulness of eazyplex^®^ SuperBug CRE test (Amplex Diagnostics) for the detection of the most important beta-lactamase genes amongst chosen Gram-negative rods.

## Materials and Methods

The tested strains were isolated from various clinical specimens, including those for rectal colonization investigation. The study involved cultures of 54 *Klebsiella* spp. and 33 *Pseudomonas aeruginosa* clinical strains. The strains with reduced susceptibility or resistant to carbapenems were specifically selected for the study purpose. The assay was also performed for 8 control strains of *K. pneumoniae*, with previously confirmed presence of genes *bla*_CTX-M1_, *bla*_CTX-M9_, *bla*_SHV-18_, *bla*_TEM-1_, *bla*_KPC_, *bla*_OXA-48_, *bla*_VIM-1_, *bla*_NDM-1_, and two control strains of *P. aeruginosa*, positive for genes *bla*_VIM-1_ and *bla*_VIM-4_. A double disc synergy test was also performed simultaneously for *K. pneumoniae* strains to detect ESBLs.

In a hospital where the study was performed, mostly ESBL-positive, NDM and VIM class metallo-beta-lactamase-positive *Klebsiella* spp. strains are isolated as well as VIM class metallo-beta-lactamase representatives of *P. aeruginosa*. Hence, eazyplex^®^ SuperBug CRE test was applied for the study purpose, covering all the mentioned beta-lactamase genes. The reference strains *K. pneumoniae* ATCC 700603 (ESBL-positive strain) and *K. pneumoniae* NCTC 13440 (*bla*_VIM_-positive strain) were used as controls in the study. The strains with the following beta-lactamase genes, KPC, NDM-1, OXA-48, as well as carbapenemase-producing *K. pneumoniae bla*_CTX-M_, *bla*_TEM_ and *P. aeruginosa bla*_VIM_ strains were genetically confirmed in our previous studies (data not shown).

The examined strains were derived from different clinical specimens: wound swab and urine—18 strains from each, blood samples—13, bronchoalveolar lavage (BAL)—12 strains, rectal swab—12, stool samples—7 strains, body cavities fluids—4, peritoneal cavity swab—2 strains and one from the blood collected via vascular catheter. (Bioethical Commission agreement no. 367/2019).

Eazyplex^®^ SuperBug CRE test (Amplex Diagnostics) was used along with Genie II instrument (OptiGene). It is based on the loop-mediated isothermal amplification (LAMP) method, and detects genes for the following beta-lactamase types and subtypes: KPC, NDM-1, VIM, OXA-48, CTX-M1, CTX-M9 and OXA-181.

To broaden the range of detected ESBLs by the genes of TEM and SHV enzymes families, PCR was performed for the entire group of strains positive in the LAMP method. The presence of the *bla*_CTX-M_ and *bla*_SHV_ genes was detected according to the methodology previously described by Jemima and Verghese [1], the presence of the *bla*_TEM_ genes according to Bali et al. method [2], *bla*_VIM_ genes were detected by Pitout et al. [3] methodology, modified by Bogiel et al. [4] and *bla*_NDM-1_ gene according to Nordmann et al. [5] procedure.

DNA from the tested strains was isolated using GeneMATRIX DNA Purification Kit (EURx). PCR was performed individually for each gene, using reaction conditions, and the primers characteristic is shown in Table 1.

**Table 1:** Primers characteristic and the conditions of each PCR used in the study

Primer name	Primer sequence (5′–3′)	Product size (bp)	PCR conditions
CTX-M F	ATGTGCAG(C/T)ACCAGTAA(A/G)GT	543	94 °C 5 min, 94 °C 1 min, 52 °C 1 min, 72 °C 1 min (35 cycles), 72 °C 10 min
CTX-M R	TGGGT(A/G)AA(A/G)TA(A/G)GT(C/G)ACCAGA		
SHV F	GGGTTATTCTTATTTGTCGC	903	
SHV R	TTAGCGTTGCCAGTGCTC		
TEM F	TTTCGTGTCGCCCTTATTCC	403	94 °C 3 min, 94 °C 45 s, 52 °C 30 s, 72 °C 1 min (35 cycles), 72 °C 3 min
TEM R	ATCGTTGTCAGAAGTAAGTTGG		
VIM F	GTTTGGTCGCATATCGCAAC	587	94 °C 5 min, 94 °C 1 min, 54 °C 1 min, 72 °C 1,5 min (30 cycles), 72 °C 10 min
VIM R	AATGCGCAGCACCAGGATAG		
NDM-1 F	GGTTTGGCGATCTGGTTTTC	624	94 °C 10 min, 94 °C 30 s, 52 °C 40 s, 72 °C 50 s (36 cycles), 72 °C 5 min
NDM-1 R	CGGAATGGCTCATCACGATC		

PCR products were separated by electrophoresis in 1.5% agarose gel (Sigma), containing Midori Green DNA Stain (ABO) and 1% TBE buffer (Bio-Rad). The conditions of electrophoresis for the ESBLs coding genes were 80 V for 85 min, while for metallo-beta-lactamase encoding genes were 90 V for 60 min.

## Results

Of the tested strains, 40 (74.1%) isolates of *Klebsiella* spp. were derived from infection cases, while 14 (25.9%) from colonization investigation. The corresponding values for *P. aeruginosa* isolates were 27 (81.8%) and 5 (18.2%). The majority of strains included into the study was isolated from adult and children patients of anaesthesiology and intensive care units—42 strains (48.3%), cardiology and internal medicine ward—15 (17.2%) and paediatrics, haematology and oncology clinic—14 (16.1%). The remaining 16 (18.4%) strains were isolated from clinical samples collected from the patients of surgery clinics. The examined strains expressed various resistance to imipenem and meropenem, and are presented in details in Table 2.

**Table 2:** Carbapenem resistance phenotypes observed amongst all the examined strains (*n* = 87)

Resistance to imipenem meropenem	*K. pneumoniae* (*n* = 50)	*K. oxytoca* (*n* = 4)	*P. aeruginosa* (*n* = 33)
IPM-R MEM-R	27	1	28
IPM-R MEM-I	14	1	5
IPM-R MEM-S	1	1	–
IPM-S MEM-R	2	–	–
IPM-I MEM-I	6	1	–

*IPM* imipenem, *MEM* meropenem, *R* resistant, *I* intermediate, *S* sensitive

The studied strains were grouped according to the species and genotypes (presence of particular set of beta-lactamase genes). For all *Klebsiella* spp. strains, the results obtained in eazyplex^®^ SuperBug CRE test and PCR were concordant. For one *P. aeruginosa* strain, a discrepancy was observed between the mentioned methods (Table 3). Concomitant ESBL and MBL genes presence has been reported in 25 K*. pneumoniae* strains and 3 *P. aeruginosa* strains. None of the examined strains possessed OXA-48 beta-lactamase gene.

**Table 3:** The comparison between the results obtained with eazyplex^®^ SuperBug CRE test and standard PCR

Species	Beta-lactamase detected	Number of the strains positive for genes presence using the following method	
		eazyplex^®^ SuperBug CRE test	Standard PCR
*K. pneumoniae*	CTX-M1, VIM	13	13
	CTX-M1, NDM	10	10
	VIM	4	4
	NDM	3	3
	CTX-M1, NDM, VIM	1	1
	CTX-M1, CTX-M9, VIM	1	1
	CTX-M1	18	18
*K. oxytoca*	VIM	4	4
*P. aeruginosa*	VIM	30	30
	CTX-M1, VIM	1	1
	CTX-M9, VIM	1	1
	CTX-M1, VIM, KPC	1	0
	CTX-M1, VIM	0	1

## Discussion

The problem of infections caused by multi-drug resistant bacteria is still growing due to clinical, epidemiological and diagnostic issues. The mentioned infections often have a fulminant course. Hence, the rapid detection of bacteria with antimicrobial resistance mechanisms is crucial since their presence may affect the effectiveness as well as the cost of the treatment. The aspect of the patient's gastrointestinal tract colonization, especially with carbapenemase-producing strains, is also of vital importance. Quick information on such a phenotype can significantly affect the limitation or complete elimination of resistant bacteria spread in the hospital environment.

CTX-M, KPC, OXA-48-like, NDM and VIM beta-lactamases are predominant resistance mechanisms amongst Gram-negative rods in Europe [6, 7]. In our hospital, there are mostly strains of *K. pneumoniae* and *P. aeruginosa* that produce ESBLs and VIM-type enzymes, respectively.

The evolution of antimicrobial resistance genes, their multiplicity and variability affect more and more reliable diagnostics by routine laboratory methods. On the other hand, standard genetic methods (e.g. typical PCR), although highly sensitive and specific, last relatively long (around 2 h, including DNA isolation procedure) when compared to the evaluated eazyplex^®^ SuperBug CRE test (Amplex Diagnostics).

To our best knowledge, eazyplex^®^ system, based on LAMP technique, was firstly described in 2014, when applied for the detection of carbapenems resistance genes amongst *Acinetobacter* spp. strains [8]. Soon after, eazyplex^®^ SuperBug complete A version of the test was mentioned. It was dedicated to Gram-negative bacteria and showed 100% sensitivity and specificity for the detection of the most common carbapenemase-coding genes [9]. Despite the mentioned features, up to day, in the available literature, there is a very few works describing the use of eazyplex^®^ SuperBug CRE test for the detection of chosen beta-lactam resistance genes [10–12].

Hinić et al. [12] used eazyplex^®^ SuperBug CRE test to detect ESBL-producing strains directly in urine specimens. The authors reached sensitivity of 100% and specificity of 97.9%, with one assay false positive and two more invalid. García-Fernández et al. [11] examined 94 strains of *Enterobacteriaceae* family (including 66 *Klebsiella* spp.) with confirmed ability to produce carbapenemases and reached 100% confirmation of their results with eazyplex^®^ test. Findlay et al. [10] analysed the results obtained for 450 strains of *Enterobacteriaceae* family and *Pseudomonas* spp. isolates. They compared the results obtained for three commercial tests: Check-direct CPE, eazyplex^®^ SuperBug complete A test, and Xpert Carba-R and did not obtain any false-positive results. In their work, eazyplex^®^ SuperBug complete A test detected correctly 83% of OXA-48 strains. Noteworthy, using eazyplex^®^ SuperBug complete A test 100% accordance of the results for the strains producing the KPC, NDM and VIM enzymes was observed. The underlined advantage of this test was the shortest performance time (20 min), comparing to the other tests. Surprisingly, the disadvantage of Check-direct CPE and eazyplex^®^ SuperBug complete A test is the lack of IMP-like enzymes genes detection.

Since, in the present study, eazyplex^®^ SuperBug CRE test (Amplex Diagnostics) was applied for clinical specimens, collected for diagnostic and epidemiological purposes, a number of issues might be underlined. The evaluated test successfully detected *bla*_VIM_ genes from two *Klebsiella oxytoca* strains derived from urine samples, each from 12-day-old twin brothers. The results were also confirmed with PCR. The bacteriuria of the neonates was estimated as 10^2^ CFU for the first sample, while only few colonies were cultured from the specimen derived from the second one. Noteworthy, the similarity of the isolated strains was investigated with MALDI Biotyper (Bruker). The PCA and MSP dendrograms, obtained for the mentioned isolates, revealed that the strains were identical when compared with the other *K. oxytoca bla*_VIM_-positive strains (data not shown).

Interestingly, eazyplex^®^ SuperBug CRE test (Amplex Diagnostics) also detected simultaneous *bla*_VIM_ and *bla*_CTX-M1_ genes carriage of four strains derived from different specimens of the same patient of the Intensive Care Unit no. 2 during over two months hospitalization. Two of them were *K. pneumoniae* strains derived from urine and BAL samples, the third was *Raoultella ornithinolytica* also cultured from BAL, the last one—*Serratia marcescens* isolated from urine sample. Strains’ identification was done using MALDI Biotyper (Bruker).

Noteworthy, with the application of eazyplex^®^ SuperBug CRE test (Amplex Diagnostics), it is possible to investigate the kinetics/course of the infection or the length of the carriage state. The first case (most probably rectal colonization leading to infection) is the patient of the Intensive Care Unit No. 2. *K. pneumoniae* strain with *bla*_NDM_ gene was detected initially from rectal swab collected for the carbapenemase-positive bacteria colonization investigation. After eight days, bacteremia was diagnosed with *K. pneumoniae* isolation from blood (however, with carbapenem-sensitive strain). Two months after, NDM-positive *K. pneumoniae* strain was isolated from the urinary tract. Simultaneously, the rectal colonization was confirmed again. One month after, patient died and NDM-positive *K. pneumoniae* strain was isolated from the blood and tissue at the autopsy investigation. Since an infection is very likely to follow colonization state, it is most probable that the same strain was found in rectal colonization, which caused bacteraemia and subsequently was also detected during autopsy investigation; however, further study is required.

The second case (an infection without earlier rectal colonization) was observed for the patient of Cardiology Clinic. Initially, rectal swab was collected for the investigation of carbapenem-resistant Gram-negative rods colonization. The result was negative, using conventional culture method. Two days after, multi-drug resistant (however, carbapenem-sensitive) *K. pneumoniae* strain was isolated from urine sample. The result of eazyplex^®^ SuperBug CRE test was *bla*_CTX-M1_ gene. Subsequently, on the same day, bacteremia was diagnosed with multi-drug resistant *K. pneumoniae* strain. For the strain, *bla*_VIM_ and *bla*_CTX-M1_ genes were detected simultaneously. Remarkably, the rectal colonization investigation was applied on the following day; however, the result was again negative. It shows that even serious infection does not have to be preceded by the colonization of the gastrointestinal tract (it is just more likely).

The present data show different probable courses of the infection, with or without initial gastrointestinal tract colonization. Regardless of the above, quick and reliable detection of the antibiotics resistant bacteria are necessary and indispensable procedures. Although sometimes expensive, their application leads to decrease of hospital infections occurrence and limits the health care associated costs. The methods chosen for this purpose should be easy to perform in each laboratory, very fast and trustworthy. Taken together, eazyplex^®^ SuperBug CRE test in general fulfils all these requirements, also for standard hospital microbiology laboratory. The test is very simple to perform; it takes no longer than 25–30 min to obtain the result. The results are repeatable and mostly in accordance with standard PCR procedure.

Worth mentioning is that the usefulness of the evaluated test is limited by the lack of possibility to detect *bla*_IMP_ genes, coding for another class of metallo-beta-lactamases, also found in Poland, e.g. amongst *Acinetobacter baumannii* strains. Taken together, the results obtained in this work confirm that eazyplex^®^ SuperBug CRE test (Amplex Diagnostics) is sensitive and reliable. Due to its ease of performance and short run times, it can be used for standard diagnostics of infection, as well as for assessment of patients’ colonization.

Based on the results, eazyplex^®^ SuperBug CRE test (Amplex Diagnostics) can be routinely used to detect rapidly the most important mechanisms of beta-lactam resistance in Gram-negative rods, for diagnostic.

## References

[1] Jemima SA, Verghese S (2008). Multiplex PCR for bla_CTX-M_ & bla_SHV_ in the extended spectrum beta-lactamase (ESBL) producing Gram-negative isolates. Indian J Med Res.

[2] Bali EB, Açık L, Sultan N (2010). Phenotypic and molecular characterization of SHV, TEM, CTX-M and extended spectrum-lactamase produced by *Escherichia coli*, *Acinetobacter baumannii* and *Klebsiella* isolates in a Turkish hospital. Afr J Microbiol Res.

[3] Pitout JDD, Gregson DB, Poirel L, McClure JA, Le L, Church DL (2005). Detection of *Pseudomonas aeruginosa* Metallo-B-Lactamases in a large centralized laboratory. J Clin Microbiol.

[4] Bogiel T, Deptuła A, Gospodarek E (2010). Evaluation of different methods for detection of metallo-beta-lactamases in clinical isolates of *Pseudomonas aeruginosa*. Pol J Microbiol.

[5] Nordmann P, Poirel L, Career A, Toleman MA, Walsh TR (2011). How to detect NDM-1 producers. J Clin Microbiol.

[6] Bevan ER, Jones AM, Hawkey PM (2017). Global epidemiology of CTX-M β-lactamases: temporal and geographical shift in genotype. J Ant Chemother.

[7] Cantón R, Akova M, Carmeli Y, Giske CG, Głupczyński Y, Gniadkowski M, Livermore DM, Miriagou V, Naas T, Rossolini GM, Samuelsen Q, Seifert H, Woodford N, Nordmann P (2012). European network on carbapenemases Rapid evolution of carbapenemases among *Enterobacteriaceae* in Europe. Clin Microbiol Infect.

[8] Vergara A, Zboromyrska Y, Mosqueda N, Morosini MI, García-Fernández S, Roca I, Cantón R, Marco F, Vila J (2014). Evaluation of a loop-mediated isothermal amplification-based methodology to detect carbapenemase carriage in *Acinetobacter* clinical isolates. Antimicrob Agents Chemother.

[9] Oseisekyere J, Govinden U, Essack SY (2015). Review of established and innovative detection methods for carbapenemase-producing Gram-negative bacteria. J Appl Microbiol.

[10] Findlay J, Hopkins KL, Meunier D, Woodford N (2015). Evaluation of three commercial assays for rapid detection of genes encoding clinically relevant carbapenemases in cultured bacteria. J Antimicrob Chemother.

[11] García-Fernández S, Morosini MI, Marco F, Gijón D, Vergara A, Vila J, Ruiz-Grabajosa P, Cantón R (2015). Evaluation of the eazyplex SuperBug CRE system for rapid detection of carbapenemases and ESBLs in clinical *Enterobacteriaceae* isolates recovered at two Spanish hospitals. J Antimicrob Chemother.

[12] Hinić V, Ziegler J, Straub C, Goldenberger D, Frei R (2015). Extended-spectrum β-lactamase (ESBL) detection directly from urine samples with rapid isothermal amplification-based eazyplex SuperBug CRE assay: proof of concept. J Microbiol Methods.

